# Gastrointestinal Inflammation and Repair: Role of Microbiome, Infection, and Nutrition

**DOI:** 10.1155/2016/6516708

**Published:** 2016-12-28

**Authors:** Helieh S. Oz, Sung-Ling Yeh, Manuela G. Neuman

**Affiliations:** ^1^University of KY Medical Center, Lexington, KY, USA; ^2^School of Nutrition and Health Sciences, Taipei Medical University, Taipei, Taiwan; ^3^University of Toronto and In Vitro Drug Safety and Biotechnology, Toronto, ON, Canada

Gastrointestinal inflammation is a complex biological response to injury as a result of different stimuli such as pathogens, damaged cells, or irritants. Symbiotic microbiome in digestive tract is considered to protect gut by removing harmful stimuli and to enhance healing process. Alteration or absence of microbiome can lead to exacerbated type 2 immunity and allergic/infectious and inflammatory complications including parasitic diseases. Thus, the microbiota regulates type 2 responses and acts as a key element in harmonizing immune responses at mucosal surfaces. While the mechanism by which microbiota regulates type 2 immunity is unclear, it is known as a strong inducer of proinflammatory T helper 17 cells and regulatory T cells (Tregs) in the intestine. The signals at the sites of inflammation mediate rapid cell recruitment and differentiation in order to remove inflammatory inducers and promote tissue homeostasis restoration. However, persistent inflammatory stimuli or dysregulation of mechanisms of the restoration can lead to chronic inflammation. Different stressors can affect immune system and increase risk for infectious diseases, such as gastritis in postinfectious irritable bowel syndrome (IBS), and vice versa, as IBS patients have increased susceptibility to develop infectious gastroenteritis. Various viral (e.g., norovirus), microbial (e.g.,* Campylobacter jejuni*,* Clostridium*,* Mycobacterium*), and parasitic agents (e.g.,* Giardia*, helminthes) are known to be involved in the development of chronic inflammatory bowel diseases. Yet, the mechanisms of action are not well known and there is no available cure. Additionally, nutritional elements, such as n-3 polyunsaturated fatty acids, antioxidants, probiotics, and prebiotics directly and indirectly modulate GI immunity. Diets high in fat change the populations of innate microbiome in digestive tract and alter signaling to the brain and satiety, leading to obesity and inflammation.

*H. pylori CagA and IL-1β*. Gastric cancer is the 3rd cause of cancer mortality globally and infection by* Helicobacter* (*H. pylori*) infecting about 50% globally. It is a main risk factor for gastric cancer by the activity of virulence factor, CagA.* H. pylori*/CagA causes chronic inflammation and triggers gastric lesions leading to cancer. IL-1*β* is linked with tumor progression including gastric cancer. Inflammatory cytokines and IL-1*β* are associated with* H. pylori* infection but link between gastric cancer and IL-1*β* was reported before. H. Arevalo-Romero et al. report a link between CagA and IL-1*β* which triggers the instigation of the epithelia to mesenchymal cells by *β*-catenin nuclear translocation. In addition, it increases the expression of Snail1 and ZEB1 and downregulates morphological changes of nontransformed epithelial cells, MCF-10A, into acini formation. CagA by itself induced MMP9 activity and cell invasion. Authors present data to support the fact that IL-1*β* and CagA trigger the *β*-catenin pathway, leading to progression of aggressive tumors.

*H. pylori and Metabolic Syndrome*.* H. pylori* infects about 50% of global population with increased risk of atherosclerosis. Similarly, patients with metabolic syndrome are at increased risk of atherosclerosis. To assess the effects of* H. pylori* infection on serum lipids, body mass index, and metabolic syndrome in old Chinese people, in a clinical trial W. Yang and C. Xuan studied association of* H. pylori* and metabolic syndrome in those who had gastroscopy exam.* H. Pylori* (+) patients had higher BMI, fasting glucose concentration, and a higher incidence of metabolic syndrome. In addition, they demonstrated that* H. pylori* infection, total cholesterol, and diabetes mellitus are significantly associated with the risk of metabolic syndrome.

*H. pylori and Nutrition*. Diet and nutrition influence* H. pylori*-associated disease result. In this review K. P. Haley and J. A. Gaddy discuss* H. pylori* ability to modify the host-immune response and to compete for the essential micronutrients. For instance, iron as well as zinc has great effect on host defense and pathogens interaction.* H. pylori has* evolved to deal with zinc stressor against microorganisms growth in human stomach by encoding multiple proteins involved in zinc efflux through its genome. In addition,* H. pylori* can change gastric flora to promote carcinogenesis, by increasing nitrosylating bacterial strains which convert nitrogen compounds in gastric fluid to carcinogens such as N-nitrosamines or nitric oxide. Authors discuss several aspects including those nutritional factors which can protect against* H. pylori* infection.

*Metagenomic and Gut Microbial Diversity*. Gut microbiome maintains nutrients metabolism and homeostasis of immune system. Metagenomics have provided the tools to explore relation between microbiome, dysbiosis, and state of diseases. A reliable methodology to profile gastrointestinal microbiome can help to reveal pathogenesis of several chronic inflammatory and infectious diseases and further to develop new preventive and therapeutic modalities. F. Benoit et al. apply profiling technology to study fecal samples from mice, cat, and a human subject. They discuss interindividual variations between these samples. Authors suggest the technique may be useful for clinical diagnostics in diet follow-up and treatments when appropriate controls are applied.

*Prealbumin/CRP Score for Predicting Metastasis*. Patients with gastric cancer have a poor prognosis with variation in their duration of survival. An appropriate prognostic technology may improve clinical care in patients. Z. Ghoreishi et al. assessed nutritional status and systemic inflammatory response of the patients before chemotherapy and present a new score system to predict metastasis. They compared prealbumin/CRP based prognostic score to compare with Glasgow prognostic score for predicting metastasis and nutritional status in patients. Authors discuss in detail statistical difference levels of prealbumin and CRP between patients with metastatic and nonmetastatic gastric cancer compared to other current methods to predict survival in this population. Future studies with large sample size will reveal the usefulness of this prognostic scoring system.

*Fish Oil, Microbiota, and Ethanol Induced Hepatic Injury*. Chronic consumption of ethanol may lead to oxidative stress, hepatic injury, alcoholic liver disease, and eventually cirrhosis. S.-C. Yang et al. investigated fish oil protective effects on hepatic structure in ethanol-fed rats based on the intestinal permeability and microbiota compared to olive oil. Ethanol induces dysbiosis, to disrupt intestinal barrier and increase permeability and endotoxemia. As was expected, liver enzymes activities, hepatic inflammatory cytokines, and plasma endotoxin levels were significantly upregulated in ethanol group with increased intestinal permeability and decreased numbers of fecal bifidobacteria (*p* < 0.05). However, these pathological effects were significantly ameliorated in those that received fish oil. The fecal* E. coli *lowered, but fecal bifidobacteria numbers were significantly increased in fish oil groups compared to olive oil. Authors conclude that dietary fish oil in ethanol consumption may normalize increased intestinal permeability and fecal microbial composition and reduce endotoxins and inflammatory cytokines and liver injury.

*Prebiotic Formula Improves GI Bacterial Flora*. Y.-L. Chen and coworkers screened healthy toddlers (18 months to 3 years) to investigate the impact of prebiotic containing formula (inulin, fructooligosaccharides, and galactooligosaccharides) on their GI microflora given 3 times a day for 6 weeks. The control formula with no prebiotics was delivered one week before and after the treatment period. Fecal samples were examined at different time points to measure the anaerobic bacteria,* Bifidobacterium *spp., and* Clostridium perfringens*. In addition, organic acids such as lactate, acetate, propionate, and butyrate were measured in the feces using high-performance liquid chromatography. The population of probiotic* Bifidobacterium *spp. significantly increased, while the total anaerobic bacteria and harmful* C. perfringens *were suppressed after 6-week administration period. Compared to that in the control period, the ratio of* Bifidobacterium *spp. to the total anaerobic bacteria significantly increased, and the ratio of* C. perfringens *to total anaerobes was significantly reduced. Furthermore, the levels of organic acids in the fecal samples significantly increased after consumption of prebiotic formula. Authors concluded that supplementation with the 3-prebiotic toddler formula may be beneficial for children's gut health.

*Xylooligosaccharides and Microbiota*. Probiotics are known to feed on prebiotics to support their growth and gut health. In a pilot trial S.-H. Lin et al. investigated the effects of short chain fatty acid (prebiotic) xylooligosaccharides and enriched rice porridge (combination cocktail) consumption additives into daily diet on the intestinal tract of human subjects. Twenty healthy subjects participated in a 6-week trial, in which half of subjects received the cocktail while the other 1/2 received placebo (rice porridge alone). Fecal samples at different time points were examined to study microflora. Investigators reveal daily consumption of the prebiotic cocktail to induce significant increases in fecal microbial counts in* Lactobacillus *spp. and* Bifidobacterium *spp., yet to decrease undesired* Clostridium perfringens* compared to placebo rice porridge. However, some changes occurring in the counts of coliforms in both groups may relate to possible favored effects by rice porridge. Authors conclude that improvement in intestinal microbiota balance supports the possible addition of prebiotic plus rice porridge into daily diet.

*Tea and Clostridium difficile Infection*. The incidence of* C. diff* infection and its mortality rate are on the rise in those susceptible. The fecal transplant has shown some benefit in the patients. Tea and its polyphenols are known best for several beneficial actions including antioxidant, anti-inflammatory, and antimicrobial effects. Contrary to beneficial reports on tea M. O. Evans II et al. reason that the antimicrobial effect of tea on gut may act similarly to undesired antibiotics in suspected individuals prone to altered gut microbiota to favor infection and* C. diff* recurrence. In a retrospective cross-sectional dietary survey investigators recruit subjects who had been diagnosed with* C. diff* infection episodes in past. From 64 enrolled patients to list their daily diet 19 had experienced recurrent episodes. A statistically significant number of patients with recurrence compared to nonrecurrence patients had used antibiotics (*p* = 0.003) or recorded tea (*p* = 0.002), coffee (*p* = 0.013), and eggs (*p* = 0.013), on 24-hour food recall. Authors conclude a possible link between tea drinking and* C. diff *recurrence to negatively affect the gut microbiota to support growth of* C. diff*. Future clinical trials are required to confirm whether tea supports the growth of* C. diff* in these recurrent patients.

*Matrix Metalloproteinases, Inhibitors, and IBD*. While the etiology of chronic inflammatory bowel disease is still under debate a series of inflammatory mediators are known to ignite the complication. Metalloproteinase (MMPs) proteolytic zinc-dependent enzymes are involved in remodeling and degradation of extracellular matrix and wound healing, as well as rheumatoid arthritis, atherosclerosis, and tumor metastasis. K. Jakubowska et al. report expression of MMPs to be distinctive in Crohn's disease compared to ulcerative colitis. The overexpression of MMPs and significantly weaker expression of the inhibitors may determine the development of IBD. Ulcerative colitis patients in particular demonstrate a significant correlation between increased MMPs expression and histopathological markers with disease progression. Authors suggest MMPs (2, 7, and 9) as potential therapeutic target and their inhibitors to reduce ulcerative colitis progression.

*Mucosal Barrier and Crohn's Disease*. The genetics, immunity, and environment factors are tightly regulating the progression of Crohn's disease in patients. Gut microbiota protects intestinal mucosa against inflammations. As dysfunction of intestinal mucosal barriers due to immune disorders and infections initiate Crohn's development, additional studies are required to explain exact protective action of intestinal mucosal barrier's in Crohn's disease. K. Wang et al. recapitulate recent findings regarding the correlations between the disorders of intestinal mucosal defenses including mechanical, chemical, immune, and biological barriers and Crohn's disease.

*Syndecan-1 and Intestinal Epithelial Inflammation*. Syndecan-1 (SDC1) is heparan sulfate proteoglycans (HSPGs) mainly expressed on the surface of epithelial cells. SDC1 is known to inhibit migration of neutrophils by downregulating chemokines and cytokines expression. Y. Zhang et al. examine the role of SDC1 in intestinal inflammatory responses using a transfected intestinal epithelial cell model. Authors conclude that suppressing SDC1 release from epithelial cells ameliorates severity of intestinal inflammation by inactivating NF-*κ*B pathway and downregulating TNF*α* expression and suggesting the use of SDC1 as a possible targeted therapy.

*Rifaximin and Mutaflor Synergy against Colitis*. A. Dembinski et al. investigated the effect of antibacterial agent, rifaximin, and/or Mutaflor, a nonpathogenic bacteria strain (*Escherichia coli* Nissle 1917), administration on the healing of acetic acid-induced colitis in rats. The authors found that rifaximin and Mutaflor had synergic anti-inflammatory and therapeutic effects in colitis. This finding implies the interaction between antibiotics coadministered with probiotics as a possible alternative choice in the treatment of colitis.

*Effectiveness and Safety of Entecavir versus Lamivudine*. This is a systematic review and meta-analysis of 1254 patients with hepatitis B viral (HBV) infections-associated acute-on-chronic liver failure. J. Yang et al. focus on 2 commonly used antiviral treatments, entecavir and lamivudine, for effectiveness and safety in HBV patients. Authors report entecavir to be significantly more effective and with higher survival rate than lamivudine in patients treated after 1 year. As for the safety, both drugs (entecavir and lamivudine) were found to be equally safe and well tolerated in patients. Further meta-analysis research needs to be conducted on adverse effects of entecavir in prolonged treatments.

*Molecular Diversity and Sapovirus Infection*.* Sapovirus*, a calicivirus, is an important pathogen involved in nonbacterial acute gastroenteritis. This study investigated the molecular diversity of* Sapovirus* in outpatients with acute gastroenteritis from the city of Nanjing in China from 2011 to 2013. H. Zhang et al. used real-time PCR with specific primers and probes targeted at conserved regions of the RNA polymerase to human* Sapovirus*. The results showed that, compared to the reference strains, different amino acid substitutions were found in the Nanjing strain. Whether these substitutions are involved in the antigenic changes or the virus fitness to hosts requires further investigation. However, this study provides some cues in the treatment of* Sapovirus*-induced enteritis in the future.

*Helieh S. Oz* *Helieh S. Oz* *Sung-Ling Yeh* *Sung-Ling Yeh* *Manuela G. Neuman* *Manuela G. Neuman*

